# Pathological Characteristics of a Patient with Severe Fever with Thrombocytopenia Syndrome (SFTS) Infected with SFTS Virus through a Sick Cat’s Bite

**DOI:** 10.3390/v13020204

**Published:** 2021-01-29

**Authors:** Masatoshi Tsuru, Tadaki Suzuki, Tomoyuki Murakami, Kumiko Matsui, Yuuji Maeda, Tomoki Yoshikawa, Takeshi Kurosu, Masayuki Shimojima, Tomome Shimada, Hideki Hasegawa, Ken Maeda, Shigeru Morikawa, Masayuki Saijo

**Affiliations:** 1 Kanmon Medical Center, Department of Hematology, Choufu Sotoura-chou, Shimonoseki City, Yamaguchi 752-8510, Japan; matsui.kumiko.sn@mail.hosp.go.jp; 2 Department of Pathology, National Institute of Infectious Diseases, 1-23-1 Toyama, Shinjuku-ku, Tokyo 162-8640, Japan; tksuzuki@nih.go.jp (T.S.); hasegawa@nih.go.jp (H.H.); 3 Kanmon Medical Center, Department of Pathology, Choufu Sotoura-chou, Shimonoseki City, Yamaguchi 752-8510, Japan; tomondenali@hotmail.co.jp; 4 Maeda Animal Hospital, 698 Takura, Shimonoseki City, Yamaguchi 751-0883, Japan; m-yuuji@mxm.mesh.ne.jp; 5 Department of Virology 1, National Institute of Infectious Diseases, 4-7-1 Gakuen, Musashimurayama City, Tokyo 208-0011, Japan; ytomoki@nih.go.jp (T.Y.); kurosu@nih.go.jp (T.K.); shimoji-@nih.go.jp (M.S.); 6 Infectious Disease Surveillance Center, National Institute of Infectious Diseases, 1-23-1 Toyama, Shinjuku-ku, Tokyo 162-8640, Japan; tomoes@niid.go.jp; 7 Department of Veterinary Science, National Institute of Infectious Diseases, 1-23-1 Toyama, Shinjuku-ku, Tokyo 162-8640, Japan; kmaeda@nih.go.jp (K.M.); s-morikawa@vet.ous.ac.jp (S.M.)

**Keywords:** severe fever with thrombocytopenia syndrome, cat, companion animals, viral hemorrhagic fever, pathology

## Abstract

A woman in her fifties showed symptoms of fever, loss of appetite, vomiting, and general fatigue 2 days after she was bitten by a sick cat, which had later died, in Yamaguchi prefecture, western Japan, in June 2016. She subsequently died of multiorgan failure, and an autopsy was performed to determine the cause of death. However, the etiological pathogens were not quickly identified. The pathological features of the patient were retrospectively re-examined, and the pathology of the regional lymph node at the site of the cat bite was found to show necrotizing lymphadenitis with hemophagocytosis. The pathological features were noted to be similar to those of patients reported to have severe fever with thrombocytopenia syndrome (SFTS). Therefore, the lymph node section was retrospectively tested immunohistochemically, revealing the presence of the SFTS virus (SFTSV) antigen. The sick cat showed similar symptoms and laboratory findings similar to those shown in human SFTS cases. The patient had no history of tick bites, and did not have skin lesions suggestive of these. She had not undertaken any outdoor activities. It is highly possible that the patient was infected with SFTSV through the sick cat’s bite. If a patient gets sick in an SFTS-endemic region after being bitten by a cat, SFTS should be considered in the differential diagnosis.

## 1. Introduction

Severe fever with thrombocytopenia syndrome (SFTS) (formerly SFTS virus, SFTSV) is caused by the *Dabie bandavirus*, which belongs to the *Bandavirus* genus (formerly *Phlebovirus* genus) of the *Phenuiviridae* family (formerly *Bunyaviridae* family). SFTS was first discovered in China [1,2] and was then reported to be endemic to Japan [3], South Korea [4], Taiwan [5], and Vietnam [6]. Patients with SFTS are usually infected with SFTSV through a tick bite such as that from *Haemaphysalis longicornis* or *Amblyomma testudinarium* [7].

The case fatality rate of patients with SFTS in Japan is reported to be approximately 30% [8,9]. There is always a risk of having SFTS for people living in SFTS-endemic regions, and it is a disease with a high fatality rate.

Recently, it has been reported that cats including cheetahs can become infected and ill with SFTSV [10,11]. Furthermore, cases of patients with SFTS infected by sick cats have also been reported [12,13]. Veterinary doctors and co-workers have been infected with SFTSV through close contact with sick cats which were virologically confirmed to be infected with SFTSV. This indicates that there is a high risk of being infected with SFTSV from sick animals through direct contact, as is the case in human-to-human SFTSV infections [14,15,16,17,18,19].

A woman who died from multiorgan failure of unknown causes was retrospectively diagnosed as having SFTS following a pathological examination. A sick cat bit her on her left hand, and symptoms appeared 2 days later. The cat also died of multiorgan failure. In this study, the clinical and pathological characteristics of the SFTS patient, who was infected by a cat positive for SFTSV infection, were revealed.

## 2. Materials and Methods

### 2.1. Patient

A woman in her fifties became ill after being bitten by a sick cat. She was retrospectively diagnosed as having SFTS as described below. Data on her clinical course, including symptoms, laboratory findings (including total blood cell (TBC) count and serum chemistry), computed tomography images, and postmortem examination, were retrospectively retrieved from her medical records.

### 2.2. Cat

Details on the clinical course and laboratory findings of the cat that bit the reported patient were retrospectively retrieved from medical records.

### 2.3. Measurement of SFTSV Genome Load with Real-Time RT-PCR in Blood

To measure copy numbers of the SFTSV S segment in sera, a quantitative one-step reverse-transcription polymerase chain reaction (RT-PCR) was performed as described previously [20].

### 2.4. Antibody Detection with Indirect Immunofluorescence Assay

An immunofluorescence assay using SFTSV-infected cells was performed to evaluate the presence of immunoglobulin (Ig)M and IgG with respect to SFTSV as described previously [21].

### 2.5. Measurement of SFTSV Genome Load with Real-Time RT-PCR in Tissues

The SFTSV copy number was determined by quantitative real-time RT-PCR on RNA samples extracted from paraffin-embedded sections (10 µm; 3×) as described previously with some modifications [3,22]. Briefly, RNA was extracted using a Pure Link FFPE RNA isolation kit (Invitrogen, Carlsbad, CA, USA), and RT-PCR was performed using a QuantiTect Multiplex RT-PCR Kit (Qiagen, Hilden, Germany) and Agilent Mx3000P system (Agilent, Santa Clara, CA, USA) according to the manufacturer’s protocol. Quantitative real-time RT-PCR amplified the N region within the S segment of the SFTSV genome. The amount of human β-actin mRNA in the RNA extracted from each section was also determined and used as an internal reference for normalization. The relative copy number of SFTSV RNA was calculated using the β-actin mRNA copy number, estimated at 1500 copies/cell, as previously described [3]. The following primers and labeled probe were used to amplify the SFTSV-N region: Primers, forward (SFTS-F2: 5′-CCCTGATGCCTTGACGATCT-3′) and reverse (SFTS-R2b: 5′-TGATTGGGTGAGGGACACAAAGTT-3′); probe 5′-(FAM) TTGCCTCGAGTCAGGGCAAAGACAA (BHQ1)-3′.

### 2.6. Pathological and Immunohistochemical Analyses

Histopathological studies of formalin-fixed and paraffin-embedded specimens were performed using hematoxylin–eosin staining. Immunohistochemical detection of the SFTSV nucleoprotein antigen (SFTSV-NP) was performed on paraffin-embedded sections, as previously described [3,22]. After deparaffinizing with xylene, sections were rehydrated in ethanol and immersed in PBS. Antigens were retrieved by hydrolytic autoclaving for 10 min at 121 °C in 10 mmol/L sodium citrate–sodium chloride buffer (pH 6.0). After cooling, the sections were immersed in PBS. Endogenous peroxidase was blocked by incubation in 0.3% hydrogen peroxide in methanol for 30 min. After washing in PBS, the sections were incubated with normal goat serum for 5 min and then with rabbit polyclonal antibody against SFTSV-NP overnight at 4 °C. After three washes in PBS, the sections were incubated with peroxidase-labeled polymer-conjugated anti-rabbit immunoglobulins (EnVision/HRP, Dako, Santa Clara, CA, USA) for 30 min at room temperature. Peroxidase activity was detected by development with diaminobenzidine containing hydrogen peroxide. Nuclei were counterstained by hematoxylin.

### 2.7. Ethical Statement

Serum samples were used for virological analysis and research purposes after obtaining written informed consent from the responsible family members. All of the protocols and procedures were approved by the Research and Ethical Committees of the National Institute of Infectious Diseases on October 2, 2017 (No. 825).

## 3. Results

### 3.1. Patient Presentation

A previously healthy woman in her fifties living in Yamaguchi prefecture in western Japan was bitten by an ill cat, which she cared for, in early summer 2016. The cat bit her on her left hand, and swelling occurred at the site of the bite. She became feverish on Day 2, with the day she was bitten considered Day 0. She showed symptoms of loss of appetite, fatigue, and vomiting on Day 3. She visited the Kanmon Medical Center and underwent a physical examination on Day 5. She was conscious. Her body temperature was 39.3 °C, her blood pressure was 122/48 mmHg, and her pulse rate was 77 beats per min. The site of the cat bite did not show any abnormal lesions except for the bite-associated scars. She did not have a history of tick bites, and did not have skin lesions suggestive of these. She had not performed any specific outdoor activities. The physician decided to treat her on an outpatient basis. Her laboratory findings on Day 5 are shown in Table 1. Most of the parameters were within a normal range (including serum chemistry) except for the presence of leukocytopenia and thrombocytopenia. Because her symptoms had worsened, she visited the hospital again on Day 8 and was hospitalized. Most of the laboratory findings at this time had become abnormal, suggesting that her physical condition had worsened further (Table 1). Her leukocytopenia had progressed, with a white blood cell count of 0.5 × 10^3^/µL, with 64% neutrophils, 32% lymphocytes, 1% monocytes, and 3% atypical cells. Liver-associated enzyme levels were also elevated. Chest computed tomography imaging revealed the presence of an enlarged lymph node in the left axillary fossa and hepatic enlargement. A bone marrow aspiration test revealed the presence of hemophagocytosis.

*Capnocytophaga canimorsus*, *Capnocytophaga canis*, or *Pasterurella* species infections, as well as toxoplasmosis, human cytomegalovirus (HCMV) infection, EB virus (EBV) infection, and viral hepatitis were listed in the differential diagnosis. A blood culture was performed before administration with anti-microbial agents. The blood tested negative with regard to blood cultures. *Pasterurella* species infection including *Pasterurella multocida* infection was also included in the differential diagnosis. However, when the patient was hospitalized, the cat bite-associated skin lesions had already healed. Therefore, the culture test could not be performed. The antibodies to *Toxoplasma gondii*, immunoglobulin (Ig) G and IgM, were measured in serum, revealing a negative reaction. Furthermore, serological tests for HCMV and EB virus (EBV) were performed because the patient showed a hemophagocytic syndrome-like disease, revealing that the infection status against HCMV and EBV was that of a past infection history. Viral hepatitis was also listed. Antibody status with regard to hepatitis A virus, hepatitis B virus, and hepatitis C virus was revealed to be negative.

Administration of methylprednisolone was initiated as treatment for the hemophagocytosis. Her consciousness deteriorated on Day 9, and her creatinine level increased, indicating kidney dysfunction. She died of multiorgan failure on Day 12 despite intensive care including respiratory support via artificial mechanical ventilation and a blood purification procedure.

### 3.2. Cat Presentation

The patient had found a cat near her house on Day-2 and noticed that it was sick. The cat seemed to have lost its appetite, was frequently vomiting, and its breathing did not seem to be regular. She took care of the cat and brought it to a veterinary hospital on Day-1. While taking care of the cat, it bit her on Day 0. The laboratory findings, TBC count, and serum chemistry of the cat are shown in Table 2. Severe leukocytopenia and thrombocytopenia were present. The data also indicated liver injury because of the abnormally high values of alanine aminotransferase (ALT) and hyperbilirubinemia. The cat died of unknown causes.

### 3.3. Dynamics of SFTSV Loads and Immunological Responses in Sera

The SFTSV copy number on Day 5 was 2.0 × 10^4^ copies/mL and reached a maximum of 2.7 × 10^9^ copies/mL on Day 11. No IgM and IgG antibodies reactive with SFTSV were detected in any sera collected during the course of the patient’s hospitalization.

### 3.4. Pathological Findings

#### 3.4.1. Gross Pathology

Gross examination on autopsy revealed mild bilateral pleural effusion (L 200 mL; R 100 mL), retention of ascites (600 mL), liver congestion, splenic congestion, and many enlarged lymph nodes (short-axis diameter of ~7 mm) throughout the mesentery, omentum, and para-aortic regions. The stomach contained 300 mL of blood clots, and gastric mucosa showed severe congestion in the absence of ulceration.

#### 3.4.2. Histopathology and Immunohistochemistry

**Figure 1:**
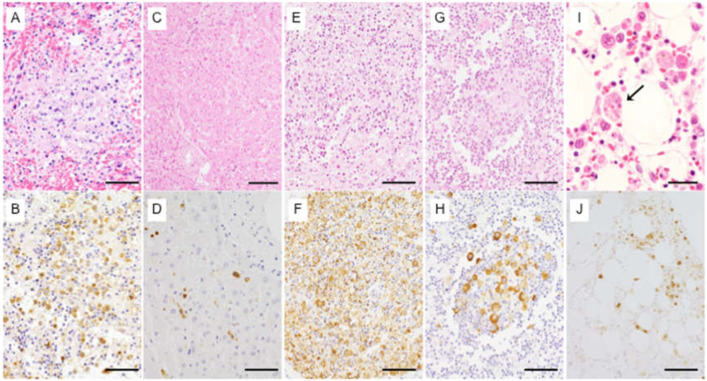
Pathological findings of the patient with SFTS who was possibly infected with SFTSV from a cat also infected with SFTSV. Histopathological and immunohistochemical evaluation of spleen, liver, bone marrow, and lymph nodes. (**A**) Spleen: atypical lymphocyte infiltration and necrotic foci. (**B**) SFTSV antigen detected in the atypical large lymphocytes. (**C**) Liver: centrilobular necrosis and hemorrhage. (**D**) SFTSV antigen detected in Kupffer cells and atypical large lymphoid cells in sinusoids in liver. (**E**) Subclavian lymph nodes: focal necrotizing lymphadenitis. (**F**) SFTSV antigen detected in necrotic debris and large lymphocytes in necrotic foci. (**G**) Sub-carinal lymph nodes: atypical large lymphocytes in follicles. (**H**) SFTSV antigen detected in the follicles of the subcarinal lymph nodes. (**I**) Bone marrow: prominent hemophagocytosis (arrow). (**J**) SFTSV antigen detected in atypical large lymphocytes. Routine hematoxylin and eosin stain: (**A**,**C**,**E**,**G**,**I**), immunohistochemical staining with anti-SFTSV NP-specific antibody: (**B**,**D**,**F**,**H**,**J**). Scale bar measurements: (**A**,**B**,**D**–**H**,**J**), 60 µm; (**C**), 100 µm; (**I**), 20 µm.

Histopathological analyses showed infiltration of numerous atypical large lymphocytes in red pulp and periarteriolar sheaths, white pulp depletion with massive nuclear debris, necrotic debris, hemophagocytosis, and congestion in the spleen (Figure 1A). Spleen, liver, multiple lymph nodes, bone marrow, kidney, thyroid, adrenal gland, and lung were positive for SFTSV antigen in the immunohistochemistry (IHC) analysis. Numerous atypical lymphocytes positive for SFTSV antigens were present in the spleen (Figure 1B). In liver, viral antigens were detected in the sinusoids amid a background of hemophagocytosis, centrilobular necrosis, hemorrhage, and mild lymphocytic inflammation around the portal tracts (Figure 1C,D). The lymph nodes in the left subclavian regions showed focal necrotizing lymphadenitis with viral antigens positive within necrotic regions (Figure 1E,F). IHC assays for *Bartonella henselae* were negative in the lymph nodes. Atypical large lymphoid cells with viral antigens were present in follicles of the mesentery and subcarinal lymph nodes (Figure 1G,H). Prominent hemophagocytosis was also evident in these lymph nodes and bone marrow (Figure 1I,J).

#### 3.4.3. SFTSV-RNA Measurement in Organs

SFTSV-RNA was detected in all organs and tissues tested, and the numbers of SFTSV-RNA copies/cell calculated using the β-actin mRNA copy number are shown in Table 3. The level of SFTSV genome copies was consistent with the IHC results.

## 4. Discussion

In the present patient, findings associated with hemophagocytosis were present in bone marrow and lymph nodes. Necrotizing lymphadenitis in lymph nodes and hemophagocytosis in bone marrow are the relatively specific and common findings in the pathology of SFTS patients [23]. The presence of necrotizing lymphadenitis and hemophagocytosis in bone marrow are reported to be positive in fatal patients with SFTS [3,22,23,24,25,26,27]. Because these factors were observed in the patient, her pathological materials were further examined for SFTSV infection.

The data in Table 3 indicate that SFTSV replicated predominantly in the spleen in this patient. SFTSV antigen was detected with IHC analyses in regional lymph nodes such as the subclavian and subcarinal lymph nodes, indicating that SFTSV replicates dominantly in the regional lymph nodes (Figure 1E,H). It was unclear whether SFTSV replicated in the non-regional lymph nodes of the patient because these lymph nodes were not tested for SFTSV antigens in the IHC analyses. Similar pathological findings have already been reported in another SFTS patient, in whom regional lymph nodes were enlarged and SFTSV was detected in these nodes but not in the non-regional lymph nodes [3]. The SFTSV antigen was detected in all organs tested in the present patient. Other reports also noted that SFTSV antigens were positive in most of the organs tested as shown in the present patient [24,25]. There were no pathological characteristics specific to this patient. All of her pathological features were similar to those reported so far [3,22,23,24,25,26,27].

However, the incubation time in the present patient was only 2 days. The SFTSV loads in blood, eye swab, saliva, rectal swabs, and urine of cats experimentally infected with SFTSV have been studied [28]. Relatively higher levels of SFTSV were demonstrated in serum, eye swabs, and saliva, but lower levels of SFTSV were found in rectal swabs and urine, suggesting high SFTSV shedding into saliva in fatal cats. The cat which bit the patient also died. Therefore, the relatively shorter incubation time does not foreclose the possibility of the cat-to-human transmission of SFTSV in this patient. It is possible that the incubation time might be shorter in a patient with SFTS if infected through the bite of a sick SFTSV-infected cat. Whether the morbidity and mortality of SFTS patients who are infected through the bite of an SFTSV-infected cat are different from those of patients infected through a tick bite should be addressed in further studies.

The clinical signs and laboratory findings of SFTSV-infected cats were characterized in 24 cats with confirmed SFTSV infection in western Japan [10]. All cats showed clinical signs of anorexia and lethargy, with fever and vomiting in 68% and 42%, respectively, and the case fatality rate was 63%. Laboratory characteristics included a TBC count indicating thrombocytopenia and leukocytopenia and elevation of serum total bilirubin, amyloid A, and CPK. A study summarizing the cases of three sick cats infected with SFTSV showed that fever and loss of appetite were the typical signs, and the TBC count revealed the presence of thrombocytopenia and leukocytopenia [12]. Another case of cat infected with SFTSV showed similar symptoms, including leukocytopenia, thrombocytopenia, elevated ALT level, and hyperbilirubinemia. The characteristics of clinical signs and laboratory findings in the cat which bit the patient in the present study were in line with those reported in SFTSV-infecting cats [10,12,13]. We were informed of the following evidence through an interview with the partner of the patient. The evidence was that the other cats reared with the cat which bit the patient showed similar symptoms and died, suggesting the disease that appeared in these cats might be a severe infectious disease.

Because this is the first case of SFTS patient possibly infected with SFTSV through a bite of a presumably SFTSV-infected cat, the incubation time reported so far for the SFTS patients possibly infected through tick bites cannot simply be applied. It was evident that the patient had suffered from SFTS after being bitten by the ill cat. These data strongly support the assumption that the patient was directly infected with SFTSV from the sick cat, although the cat was not specifically diagnosed as having SFTSV infection virologically.

A male veterinarian in western Japan was infected with SFTSV by a sick cat [12]. The patient had taken care of sick mammals, including three cats suspected of having SFTSV infection. A partial nucleotide sequence of the SFTSV genome amplified with RT-PCR from a blood sample of the patient was identical to that detected in the cats. According to the report, the partial SFTSV genome sequences detected in the three cats were identical.

The present patient suffered from SFTS-like disease 2 days after being bitten by a sick cat. However, SFTS was not included in the differential diagnosis because it was not well known that sick cats could be a vector for the transmission of SFTSV to humans.

The pathological examination following the patient’s autopsy made it possible to diagnose her retrospectively as having SFTS. The first patient with SFTS diagnosed in Japan was autopsied to determine the cause of death [3]. The patient was diagnosed as having SFTS retrospectively by isolation of SFTSV from blood samples. Pathological examinations including IHC for SFTSV infections were performed to clarify the characteristics of SFTSV infections in humans, indicating that the presence of necrotizing lymphadenitis in regional lymph nodes and hemophagocytosis in bone marrow were characteristic of SFTS [3]. The present study confirms the importance of performing autopsy on patients dying of unknown causes. Domestic cats live with humans, and humans have close contact with their cats. Some cat owners let their domestic cats go outdoors, which can result in an increased risk of the cats being infected with SFTSV through a tick bite.

The present study revealed that there is a risk of being infected with SFTSV not only through tick bites but also through close contact or a bite from a sick cat infected with SFTSV. If a patient gets sick in an SFTS-endemic region after a cat bite, SFTS should be considered in the differential diagnosis. Further, cat owners and veterinary personnel in SFTS-endemic regions should be advised to treat sick cats with careful infection control measures and not to be bitten by or have direct or close contact with cats afflicted by illnesses of unknown causes.

## Figures and Tables

**Table 1:** Sequential laboratory data of the patient with SFTS.

Categories	Normal Range	Day 5	Day 8	Day 9	Day 11
Total blood cell counts					
WBC (×10^3^ cells/µL)	3.3–8.6	1.9	0.5	0.5	3.1
Platelets (×10^3^ cells/µL)	158–348	133	67	52	49
RBC (×10^6^ cells/µL)	3.86–4.92	4.50	4.67	4.61	5.14
Serum chemistry					
TP (g/dL)	6.6–8.1	6.8	6.1	5.4	5.7
ALB (g/dL)	4.1–5.1	4.1	3.5	3.1	2.9
TB (mg/dL)	0.40–1.50	0.64	0.71	0.58	1.85
AST (U/L)	13–30	23	494	1210	3784
ALT (U/L)	7–23	14	169	376	961
LDH (U/L)	124–222	187	940	1584	5021
ALP (U/L)	106–322	189	201	220	425
γ-GTP (U/L)	9–32	15	23	26	85
BUN (mg/dL)	8.0–20.0	14.3	17.6	NT	31.4
CRE (mg/dL)	0.46–0.79	0.57	0.58	0.52	0.90
Na (mmol/L)	138–145	133	134	133	130
K (mmol/L)	3.6–4.8	4.2	3.6	3.6	4.5
Cl (mmol/L)	101–108	96	93	96	94
CRP (mg/dL))	0.00–0.14	0.04	0.03	0.06	0.04
PT (second)	10.5–15.5	NT	14.2	14.4	15.0
APTT (second)	30.0–40.0	NT	65.3	76.7	82.1
Fibrinogen (mg/dL)	150.0–450.0	NT	229.0	NT	203.0
D-dimer (µg/mL)	0.00–0.40	NT	11.51	12.88	9.07

Abbreviations: SFTS, severe fever with thrombocytopenia syndrome; WBC, white blood cells; RBC, red blood cells; TP, total protein; ALB, albumin; TB, total bilirubin; AST, aspartate aminotransferase; ALT, alanine aminotransferase; LDH, lactate dehydrogenase; ALP, alkaline phosphatase; γ-GTP, gamma glutamyl transpeptidase; BUN, blood urea nitrogen CRE, creatinine; Na, sodium; K, potassium; Cl, chloride; CRP, C-reactive protein; PT, prothrombin; time; APTT, activated partial thrombin time; NT, not tested.

**Table 2:** Total blood cell counts and serum chemistry of the sick cat.

Categories	Normal Range	Values
Total blood cell counts		
WBC (×10^3^ cells/µL)	2.07–17.02	0.87
Platelet (×10^3^ cells/µL)	151–600	12
RBC (×10^6^ cells/µL)	6.54–12.20	9.76
Serum chemistry		
TP (g/dL)	5.2–8.2	7.7
ALB (g/dL)	2.2–3.9	2.6
ALT (U/L)	12–130	234
ALP (U/L)	14–192	<10
TB (mg/dL)	0–0.9	5.3
BUN (mg/dL)	13–33	25
CRE (mg/dL)	0.6–1.6	1.1
Na (mmol/L)	150–165	149
K (mmol/L)	3.7–5.9	3.1
Cl (mmol/L)	115–156	110
Urinalysis		
Protein	Negative	++
Hematuria	Negative	+++

Abbreviations: WBC, white blood cells; RBC, red blood cell; TP, total protein; ALB, albumin; ALT, alanine aminotransferase; ALP, alkaline phosphatase; TB, total bilirubin; BUN, blood urea nitrogen CRE, creatinine; Na, sodium; K, potassium; Cl, chloride.

**Table 3:** Distribution of SFTS viral NP antigen and SFTS viral RNA in formalin-fixed and paraffin-embedded tissue sections from the autopsied patient.

Tissue Section	Measurement of SFTSV Genome with Quantitative Real-Time Reverse Transcription PCR Assay			IHC
	SFTSV RNA (Copies/Reaction)	β-Actin (Copies/Reaction)	Copies/Cell *	(SFTSV-NP)
Spleen	4.73 × 10^4^	7.96 × 10^3^	8.91 × 10^3^	++++
Liver	2.53 × 10^3^	5.81 × 10^3^	6.53 × 10^2^	++
Adrenal gland	6.08 × 10^1^	1.98 × 10^3^	4.62 × 10^1^	++
Bone marrow	1.23 × 10^0^	4.36 × 10^1^	4.24 × 10^1^	++
Left subclavian lymph node	2.08 × 10^2^	8.83 × 10^3^	3.54 × 10^1^	++
Lung	9.52 × 10^1^	5.85 × 10^3^	2.44 × 10^1^	+
Thyroid	4.02 × 10^1^	4.80 × 10^3^	1.26 × 10^1^	+
Mesenteric lymph node	3.88 × 10^1^	4.99 × 10^3^	1.17 × 10^1^	++
Kidney	4.81 × 10^1^	8.51 × 10^3^	8.48 × 10^1^	+
Subcarinal lymph node	1.90 × 10^1^	6.19 × 10^3^	4.61 × 10^1^	++

Abbreviations: SFTS, severe fever with thrombocytopenia syndrome; NP, nucleoprotein antigen; SFTSV, SFTS virus; IHC: immunohistochemistry. * (Copies/cell): copy/cell = SFTSV/β-actin × 1500.

## Data Availability

The data presented in this study are available in the article.
